# Occlusal Changes with Clear Aligners and the Case Complexity Influence: A Longitudinal Cohort Clinical Study

**DOI:** 10.3390/jcm12103435

**Published:** 2023-05-12

**Authors:** Vanessa Marcelino, Sofia Baptista, Sandra Marcelino, Maria Paço, Duarte Rocha, Maria dos Prazeres Gonçalves, Rui Azevedo, António Sérgio Guimarães, Gustavo Vicentis Oliveira Fernandes, Teresa Pinho

**Affiliations:** 1 UNIPRO—Oral Pathology and Rehabilitation Research Unit, University Institute of Health Sciences (IUCS), Cooperativa de Ensino Superior Politécnico e Universitário (CESPU), 4585-116 Gandra, Portugal; 2 Centro de Investigação de Montanha (CIMO), Instituto Politécnico de Bragança, Campus de Santa Apolónia, 5300-253 Bragança, Portugal; 3 TOXRUN—Toxicology Research Unit, University Institute of Health Sciences (IUCS), Cooperativa de Ensino Superior Politécnico e Universitário (CESPU), 4585-116 Gandra, Portugal; 4 Laboratory of Neuroimmune Interface of Pain Research, Faculdade São Leopoldo Mandic, Campinas 13045-755, Brazil; 5 Periodontics and Oral Medicine Department, University of Michigan School of Dentistry, Ann Arbor, MI 48109, USA; 6 Institute for Molecular and Cell Biology, i3S-Instituto de Investigação e Inovação em Saúde, Universidade do Porto, 4200-135 Porto, Portugal

**Keywords:** clear aligners, masticatory function, occlusal contacts, occlusal area, facial biotype

## Abstract

Background: Clear aligners (CA) are used 22 h daily, creating a bite-block effect. This work aims to (i) analyze occlusal changes before the beginning of treatment, after the first set of CA and after the use of additional aligners; (ii) compare planned occlusal contacts with the ones obtained after the first set of CA; (iii) analyze the occlusal changes occurred after reaching the orthodontic goals after 3 months of using CA only at night; (iv) evaluate and characterize which tooth movements did not allow the treatment to be completed at the end of the first set of aligners, and finally (v) verify the possible relation between the changes in occlusal contact and areas and parameters such as case complexity and facial biotype. Materials and Methods: A quantitative, comparative, and observational longitudinal cohort study design was implemented to evaluate the clinical data and the complexity levels of cases receiving CA. A non-probabilistic and convenience sample of 82 individuals was recruited. The orthodontic malocclusion traits were classified as simple, moderate, or complex corrections based on the basis of the Align^®^ recommendations with the Invisalign^®^ evaluation tool. According to the Invisalign^®^ criteria, patients need only one complex problem for their case to be classified as complex. Meshlab^®^ v. 2022.02, ClinCheck^®^ version Pro 6.0, My-Itero^®^ version 2.7.9.601 5d plus, and IBM^®^ SPSS Statistics software (Statistical Program for Social Sciences), version 27.0 for Windows were the software^®^ used. Results: A statistically significant decrease in area and occlusal contacts number were observed from before the start of orthodontic treatment (T0) to the end of treatment (T1). The changes in the occlusal area (from T0 to T1) were statistically different between hyperdivergent (28.24 [15.51–40.91]) and hypodivergent (16.23 [8.11–24.97]) biotypes (*p* = 0.031). A significant difference between the hyperdivergent (4.0 [2.0–5.0]) and normodivergent (5.5 [4.0–8.0]) group was found in T1 for the anterior contacts (*p* = 0.044). Anterior contacts obtained were significantly higher than the planned (*p* = 0.037) Between T1 and T2 statistically significant increases of occlusal areas, posterior and total contacts were observed. Conclusions: Occlusal contact and area were decreased, either at the end of the first set or after the use of additional aligners. Anterior occlusal contacts obtained were higher than planned as opposed to posterior occlusal contacts obtained. The hardest tooth movements to achieve to complete the treatment were distalization, rotation, and posterior extrusion. After completing orthodontic treatment (T1) to 3 months after (T2) using additional aligners only at night, posterior occlusal contacts were significantly increased, which could be due to the natural settling of the teeth in this period.

## 1. Introduction

Orthodontics has been one of the areas in Dentistry seeing rapid development. The use of aesthetic brackets, lingual orthodontic appliances, or clear aligners (CA) appeared to hide the use of metal brackets [1]. With regards to clear aligner treatment (CAT), the use of a planning software has allowed higher predictability. However, some of the main disadvantages of these treatments are the limited control of root movement and intermaxillary correction [1].

Planning an orthodontic treatment with CA is performed differently from conventional fixed treatment, although the basic orthodontics concepts remain the same [2]. It is necessary to consider that, regardless of the technique used, several occlusal changes happen due to tooth movement during orthodontic treatment [3,4,5]. Therefore, developing a balanced occlusion for allowing a proper function is one of the issues that needs to and considered when implementing an individualized treatment plan [6].

The most mentioned limitation in the literature refers to CAT as less effective in achieving occlusal contacts than fixed appliances. Controlling the buccolingual tipping of posterior teeth is also difficult, due to the creation of an artificial interference linked to the use of the CA, referred as “bite-block effect” in the literature [1,7,8]. The failure to achieve stable and solid occlusal contacts has been discussed as one of the reasons for the higher relapse rate associated with CAT [1,7]. This lack of posterior contacts can resolve itself, naturally after the conclusion of the treatment, called settling of occlusion [9]. Depending on the clinical orthodontic situation, the proposed treatment plan often implements only one set of aligners [10,11]. However, this is not always feasible and in order to meet, with CA, esthetic and functional treatment objectives with often further additional aligners are required to attain all orthodontic treatment (OT) objectives [12,13,14].

Although the algorithm in the ClinCheck^®^ software (Invisalign^®^ system) determines the tooth movements necessary to obtain the desired final occlusion, several experts recommend planning an overcorrection due to possible relapses [13,15]. In addition to this, the orthodontist must perform excellent vertical control taking into consideration the vertical characteristics of the patient [16,17]. For instance individuals with a hyperdivergent biotype are usually have a more flaccid and weakened facial musculature [18,19]. In those cases, the orthodontist must provide greater control of vertical growth during orthodontic mechanics, mainly due to the possibility of the posterior sectors extrusion, aggravating the vertical tendency [20]. The hypodivergent biotype, on the other hand, is associated with greater muscle strength, requiring stronger opening biomechanics and, in these cases, avoiding the tendency for posterior sectors to intrude [17].

It is not sufficiently studied how the number of occlusal contacts and area evolve during a CAT, taken into account the different case complexities. This real issue needs to be understood in order to achieve the best results from CAT. Thus, this article aims to (i) analyze occlusal changes before the beginning of treatment, after the first set of CA and after the use of additional aligners; (ii) compare planned occlusal contacts with the ones obtained after the first set of CA; (iii) analyze the occlusal changes occurred after reaching the orthodontic goals after 3 months of using CA only at night; (iv) evaluate and characterize which tooth movements did not allow the treatment to be completed at the end of the first set of aligners, and finally (v) verify the possible relation between the changes in occlusal contact and areas and parameters such as case complexity and facial biotype.

## 2. Materials and Methods

### 2.1. Study Design

This study is a quantitative, comparative, and observational longitudinal cohort study design. It followed the Declaration of Helsinki (1975, updated 2013), it was designed in accordance with CONSORT (http://www.consort-statement.org/, accessed on 16 December 2019), and it was approved by the local Ethics Committee (protocol 1/CE-IUCS/2019). After the explanation, evaluation, agreement, and signing of the Informed Consent, the patients were enrolled.

### 2.2. Samples and Eligibility Criteria

A non-probabilistic and convenience sample was recruited from cases with complete permanent dentition (excluding third molars). They were undergoing orthodontic CAT in a private clinic under the supervision of a double specialist in Orthodontics and Odontopediatrics, also an Invisalign Diamond Provider (T.P.). This study’s inclusion and exclusion criteria are reported in Table 1. Our study sample consists of eighty-two individuals (n = 82) that completed the first set of aligners (regardless of whether their planned orthodontic objectives were achieved).

### 2.3. Occlusal Measurements

After analyzing the clinical records, each participant’s characteristics were collected at the beginning of CAT (T0) and evaluated, namely gender and age. Cephalometric tracing, overbite, overjet, and facial biotype (FMA) were also measured and assessed. The facial biotype classification was performed taking into account the FMA angle (formed by the Frankfurt plane and the mandibular plane), with participants being classified as hypodivergent when values were equal to or below 22 degrees, normodivergent 23 to 27 degrees and hyperdivergent with values equal to or above 28 degrees [21,22].

The evaluation was performed for all individuals at different time-points: (T0) before starting the CAT (T1) at the end of the orthodontic treatment when the orthodontic goals have been achieved (either at the end of the first set of CA or after the use of additional aligners to complete the treatment) and (T2) 3 months after the end of the first set and using additional aligners only at night. At all these time-points occlusal contacts and areas were evaluated. For each participant, intra-oral images from iTero^®^ and photographs of the oral cavity were first obtained and then treated with the ClinCheck^®^ software to obtain the treatment plan. The needed PLY file was recovered from the intraoral scanner as follows: selected in the Itero^®^ software: “open Shell”, “arches combined (arches locked in bite relation)”, and “PLY (color)”. Those PLY files were then used and analyzed by the Meshlab^®^ software to obtain the occlusal areas. Those procedures where repeated at T0, T1 and T2 for all individuals. The occlusal area calculated through Meshlab^®^ took into consideration the distance between inter-arch occlusal contacts equal or less than 0.2 mm, as previously mentioned in the literature [23,24]. The areas that fit these parameters appear as marked with a red coloration.

Cephalometric measurements and above-mentioned data were organized in an Excel file. These were later used for statistical analysis. MeshLab^®^ software was used to obtain occlusal areas from T0, T1 and T2. To minimize errors of recollection, a classical clinical occlusion analysis using articulating paper was also performed. This additional assessment was used to ensure accuracy of the digital models and bite registration. Only the planned occlusal contacts were treated through ClinCheck^®^ software. Any opposing pair of teeth, maxillary and mandibular were counted as one occlusal contact. Counting the pairs was performed on the lower arch. The maximum expected number of occlusal contacts in a twenty-eight-tooth dentition is fourteen. The maximum of posterior occlusal contacts is eight, and the maximum of anterior occlusal contacts is six. Third molars were excluded for the areas and occlusal contact measurements.

### 2.4. Orthodontic Intervention

The individuals were instructed to use each aligner for as many hours as possible (20–22 h/day) and only to remove them to eat and perform oral hygiene. The aligners were changed every seven days, as recommended by Align^®^ protocols. The control consultations were carried out every two months. Additional aligners are recommended in two distinct situations: (a) when orthodontic goals have not been reached in the context of the first Clincheck^®^ orthodontic objectives or (b) to use only at night, in cases where the OT goals were attained, to improve the occlusal contacts and refine any minor orthodontic details needed to enhance occlusion.

### 2.5. Clinical Assessment—Complexity of the Case

Based on the clinical data (clinical photographs, radiographs, and digital images through intra-oral scanner), the complexity levels of cases receiving CAT were independently assessed by two authors (V.M. and S.B.). In cases of discrepancy, a third researcher was consulted (T.P.). For this purpose, an online assessment tool available in Invisalign^®^ Doctor Site was used [11]. The orthodontic malocclusion traits were classified as simple, moderate, or complex corrections based on the Align^®^ recommendations with the Invisalign^®^ evaluation tool. According to the Invisalign^®^ criteria, patients only need one complex problem for their case to be classified as complex. The evaluator of case complexity is composed of a series of clinical conditions that lead to a final classification: simple, moderate, or complex. This classification considers different parameters: type of dentition, need for surgery, the amount of spacing, crowding, rotations, narrow arches, posterior cross-bite, anteroposterior correction, anterior cross-bite, anterior open bite, deep bite, and need to extraction. Each of these parameters has sub-parameters which were also evaluated.

### 2.6. Sequenced and Tooth Speed Movement Control

A different sequencing model of tooth movement can be decided by the responsible specialist. This kind of sequencing is performed according to Bollen et al. and Clement et al., If less movement is built into each aligner, then the number of aligners needed for each required movement will be increased. This different sequencing intends to fractionate the desired movement, to ensure its success. The aligners were changed weekly to ensure the planned complex movement [25,26].

### 2.7. Statistical Analysis

Data analysis was performed using the IBM^®^ SPSS program (Statistical Program for Social Sciences), version 27.0 for Windows. Descriptive statistics were performed to estimate frequencies, percentages, means, medians, standard deviations, minima, and maxima. The Shapiro-Wilk test was used to assess the normality of the variables under study. Since they did not follow a normal distribution, a non-parametric analysis was applied. Therefore, to compare the areas and the number of occlusal contacts, anterior and posterior, between T0 and T1, the non-parametric Wilcoxon Signed-Rank test was used. The contact area and the number of anterior and posterior occlusal contacts at T0 and T1 were compared, for the different combinations of biotypes, using the non-parametric Kruskal-Wallis test, followed by Dunn’s test with Bonferroni correction to understand which ones differed significantly from each other. Kruskal-Wallis effect size and respective confidence intervals were calculated in R [27] based on Tomczak and Tomczak (2014), using boostrapping with 1000 replications. It was considered <0.06 (small effect), <0.14 (moderate effect) and ≥0.14 (large effect). Fisher’s exact test was used to assess the relationship between the complexity of the case and the facial biotype. Friedman test was used to evaluate the occlusal contacts and areas between T0, T1, and T2.

## 3. Results

### 3.1. Sample Characteristics

According to the inclusion and exclusion criteria (Table 1), the final sample included participants ages ranging from 12 to 49 years old (average 23.67 ± 10.17), 54 females (65.9%), and 28 males (34.1%). Regarding the facial biotype, 34 presented a normodivergent biotype (41.5%), 27 hyperdivergent (32.9%), and 21 (25.6%) hypodivergent. Most participants showed a normal overbite (85.4%) and normal overjet (76.8%). The clinical study of the sample revealed 42 cases with an open bite (51.2%), 28 with a deep bite (34.1%), and finally, 12 presented a normal bite (14.6%).

The 82 subjects in the sample completed the first set of aligners. Of these, only 54 achieved the proposed orthodontic goals and finished their orthodontic treatment, and the last 28 did not reach the planned orthodontic goals despite finishing the first set of aligners, so not completing the orthodontic treatment.

### 3.2. The Occlusal Contact and Area at T0 and T1 of the 82 Subjects

**Figure 1:**
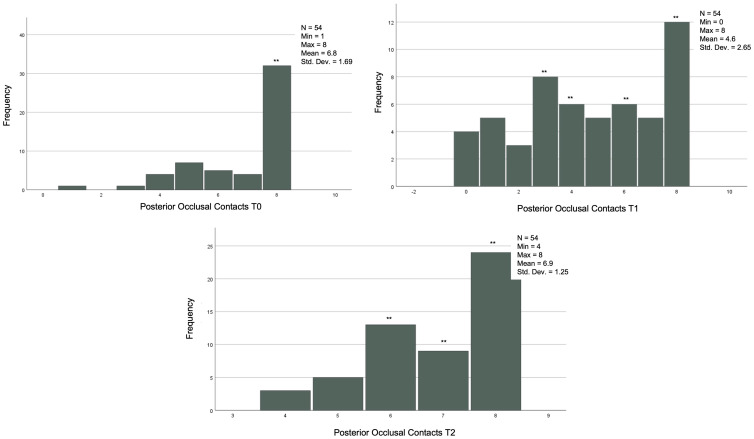
Analysis posterior occlusal contacts. T0, T1 and T2 of the 54 individuals that achieved the proposed orthodontic goals on the first set of aligners. Related Sample Friedmand’s Two-Way Test. ** Significant difference from posterior contacts T0 and T1 (*p* < 0.001) and T1 and between posterior contacts T1 and posterior contacts only with night use (*p* < 0.001). (T0)—before the start of orthodontic treatment, (T1) the end of treatment and (T2) 3 months after using additional aligner only at night.

**Figure 2:**
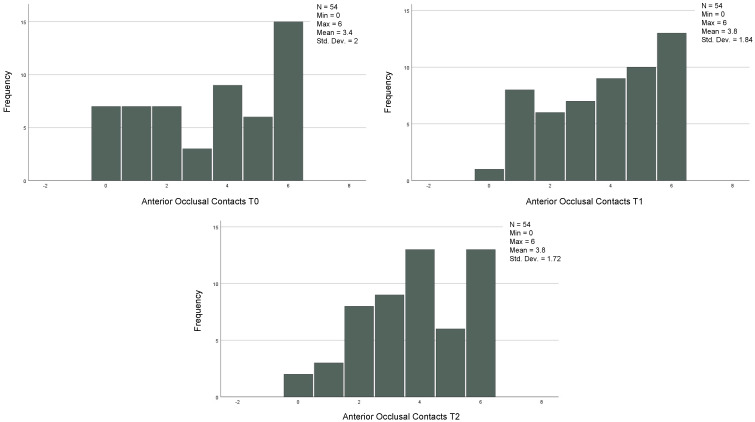
Analysis anterior occlusal contacts. T0, T1 and T2 of the 54 individuals that achieved the proposed orthodontic goals on the first set of aligners. (T0)—before the start of orthodontic treatment, (T1) the end of treatment and (T2) 3 months after using additional aligner only at night. Related Sample Friedmand’s Two-Way Test.

The descriptive statistics referring to the continuous variables are reported in Table 2 and Figure 1 and Figure 2.

### 3.3. Occlusal Area and Number of Anterior and Posterior Contacts before and after the First Set of CAT of the 82 Subjects

Table 3 presents the obtained results when comparing the occlusal area and the number of anterior and posterior contacts at T0 and at T1. We found that there are statistically significant differences in terms of the occlusal area (Z = −5.59; *p* < 0.001), with a decrease after the first set of the CA (T1). Similarly, the number of posterior occlusal contacts was significantly reduced (Z = −7.39; *p* < 0.001) after the first set of the CA (T1). Finally, regarding the number of anterior occlusal contacts, there were statistically significant differences (Z = −3.81; *p* < 0.001), showing an increase in the number of contacts after the first set of CAT (T1).

### 3.4. Number of Programmed Anterior and Posterior Contacts and Those Obtained after the First Set of CAT of the 82 Subjects

Evaluating the obtained results (Table 4), posterior contacts were found to be statistically different after the first set of CA (Z = −6.52; *p* < 0.001), attesting that the number of contacts in T1 after the first set of CA, were lower than programmed. In addition, the number of verified anterior occlusal contacts in T1 were significantly higher than programmed (Z= −7.45; *p* < 0.001).

### 3.5. Occlusal Area and the Number of Anterior and Posterior Contacts, according to Facial Biotype on the 82 Subjects

The comparison between the three facial biotypes (Table 5) showed a decrease in the occlusal area at the end of the first series of CA in all 82 cases and regarding anterior contacts obtained at T1. The posterior contacts before and after the end of the first set of CA presented no statistically significant differences between the groups. A low effect size was obtained for the occlusal area as well as for the anterior and posterior occlusal contacts at T0. At T1, a moderate effect size was found only for the occlusal areas.

### 3.6. Comparison between Numbers of Anterior and Posterior Contacts at T0, T1, and T2 in the Group That Achieved the Orthodontic Goals after the First Set of CA

**Figure 3:**
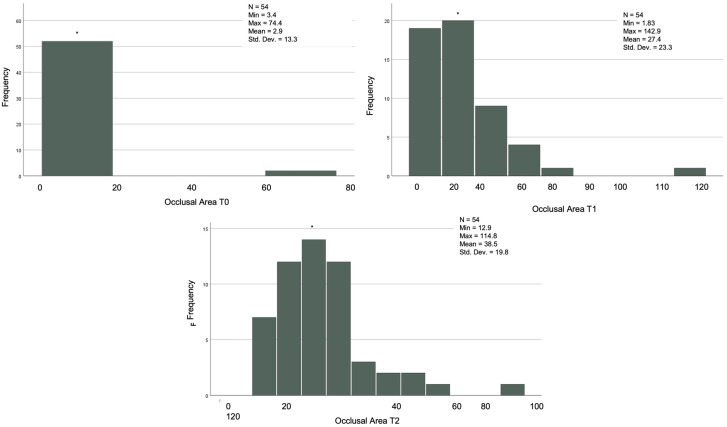
Analysis of occlusal areas at T0, T1 and T2 of the 54 individuals that achieved the proposed orthodontic goals on the first set of aligners. Related Sample Friedmand’s Two-Way Test. * Significant difference from oclusal area T0 and T1 (*p* < 0.001) and occlusal area T1 and occlusal area only with night use (*p* = 0.001). (T0)—before the start of orthodontic treatment, (T1)—the end of treatment and (T2)—3 months after using additional aligner only at night.

**Figure 4:**
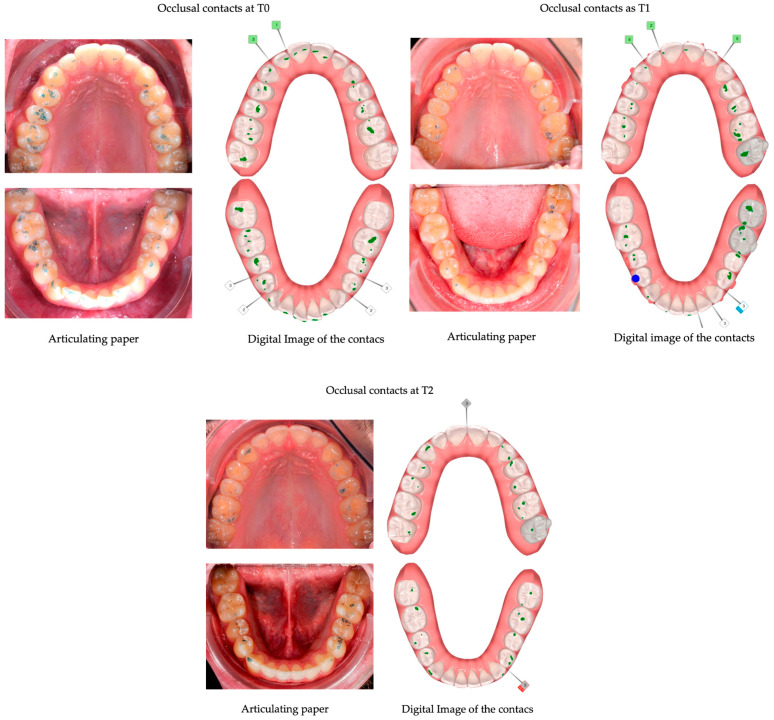
Comparison of the alterations of occlusal contacts during clear aligner treatment, obtained through intra-oral scanning and articulating paper. (T0)—before the start of orthodontic treatment, (T1)—the end of treatment and (T2)—3 months after using additional aligner only at night.

**Figure 5:**
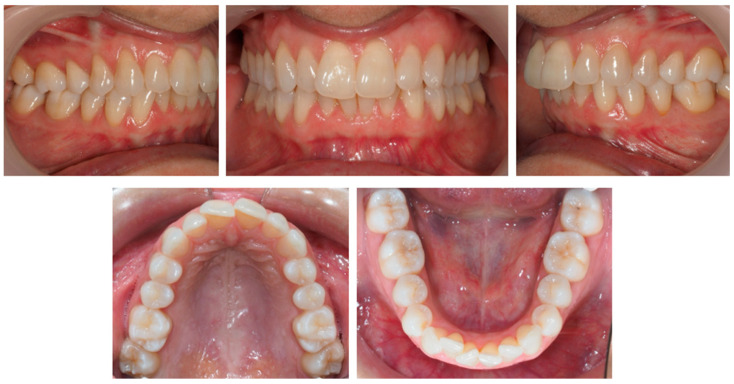
Clinical study 2—Intra-oral images at T0. (T0)—before the start of orthodontic treatment.

**Figure 6:**
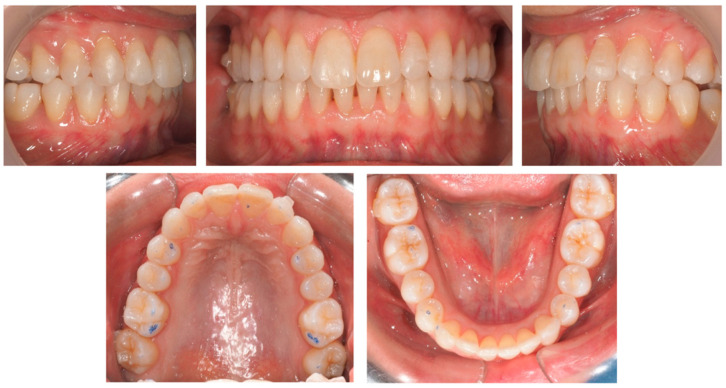
Clinical study 2—Intra-oral images at T1. Case concluded after the first set of clear aligner, achieving the orthodontic goals, with visible lack of posterior occlusal contacts. (T1)—the end of treatment.

**Figure 7:**
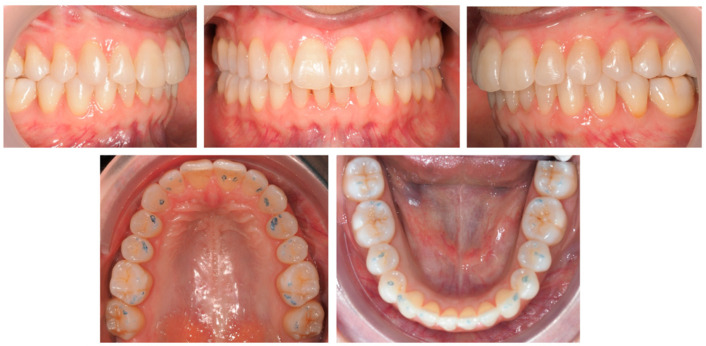
Clinical study 2—Intra-oral images at T2, with visible settling of posterior occlusal contacts, 3 months after the end of the treatment with clear aligner and using only at night.

**Figure 8:**
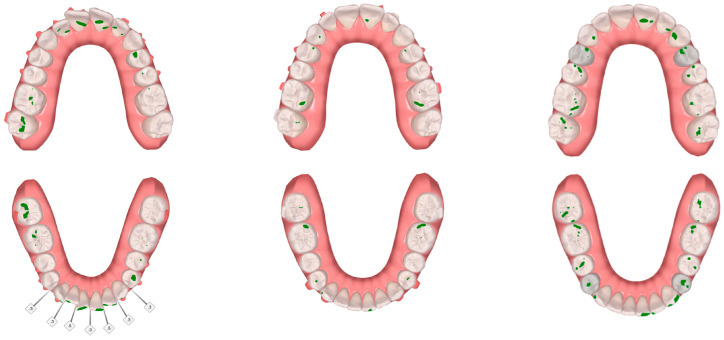
Clinical study 2—Occlusal contacts obtained at T0, T1 and T2, though the ClinCheck^®^ software version Pro 6.0. (T0)—before the start of orthodontic treatment, (T1) the end of treatment and (T2) 3 months after using additional aligner only at night.

**Figure 9:**
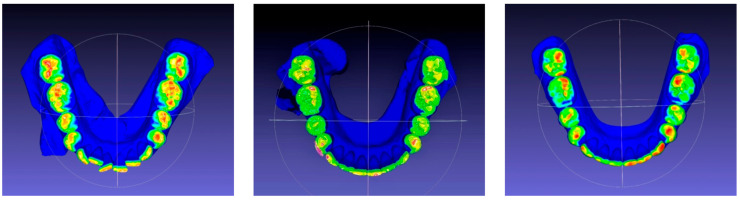
Occlusal areas obtained at T0, T1 and T2 through Meshlab^®^ software version 2022.02.

In the 54 cases that reached the orthodontic goals and therefore completed the treatment after the first series of CA, the posterior, anterior, and total contacts were assessed at the three time-points (T0, T1 and T2) (Table 6). The posterior contacts were reduced from T0 to T1 (*p* < 0.001) and increased from T1 to T2 (*p* < 0.001) (Figure 1). Regarding anterior contacts (Figure 2), the variation between T0, T1, and T2 was minimal, without reaching statistical significance. The total contacts however decreased significantly from T0 to T1 (*p* = 0.014) and increased from T1 to T2 (*p* = 0.004). Regarding occlusal areas, statistical significance was obtained at all time-points (*p* < 0.001) (Figure 3). The comparison of occlusal contacts obtained through digital image and articulating paper is represented in Figure 4. Occlusal contacts and areas of a clinical case are represented from Figure 5, Figure 6, Figure 7, Figure 8 and Figure 9.

### 3.7. Case Complexity

Case complexity of each individual patient was assessed at the beginning of the treatment. Of the 82 individuals that underwent CAT, 46 were classified as being of moderate complexity (56.1%), 23 were considered simple (28%), and 13 were classified as complex cases (15.9%). The movements justifying the classification of case complexity are enumerated in Table 7. The number of CA used during the first set of aligners ranged from a minimum of 13 to a maximum of 77 (32.63 ± 12.97). At the end of the first set of CA, 54 cases achieved the planned orthodontic goals (65.9%) and thus finished the treatment. Of the 28 uncompleted cases (34.1%), 8 were classified as complex (28.6%), and 20 were moderate (71.4%). Of those 28 unfinished cases, the movements that did not allow treatment completion as well as their degree of movement were reported in Table 8 and Table 9.

### 3.8. Number of Additional Aligners Needed to Complete the Orthodontic Case

In the 28 cases that did not reach the orthodontic goals and therefore did not complete the treatment at the end of the first set of aligners, additional aligners were required to do so. The number of additional aligners (AA) is described in Table 10.

### 3.9. Comparison between Numbers of Anterior and Posterior Contacts at T0, T1, and T2 in the Group That Achieved the Orthodontic Goals Only after the Use of Additional Aligners (28 Individuals)

**Figure 10:**
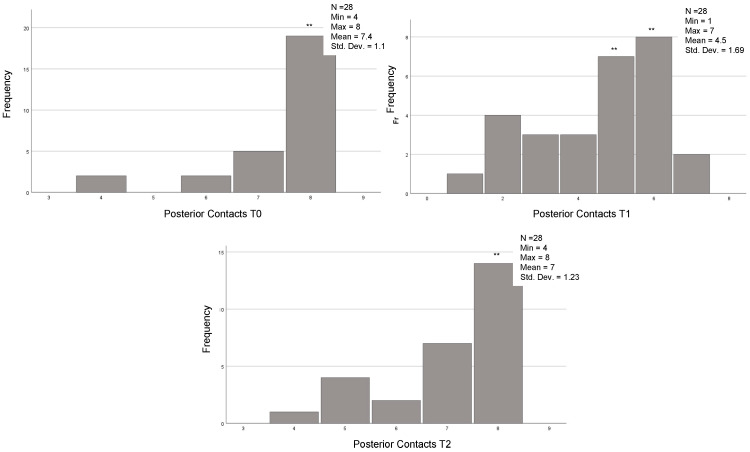
Analysis posterior occlusal contacts. T0, T1 and T2 of the 28 individuals that used AA. Related Sample Friedmand’s Two-Way Test. ** Significant difference from posterior contacts T0 and T1 (*p* = 0.000) and T1 and between posterior contacts T1 and posterior contacts only with night use (*p* = 0.000). (T0)—before the start of orthodontic treatment, (T1) the end of treatment and (T2) 3 months after using additional aligner only at night.

**Figure 11:**
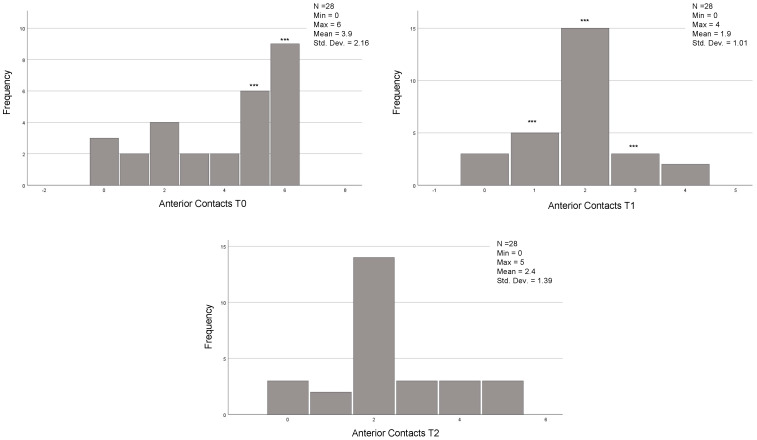
Analysis of anterior occlusal contact at T0, T1 and T2 of the 28 individuals that used AA.Related Sample Friedmand’s Two-Way Test. *** Significant difference from anterior contacts T0 and T1 (*p* = 0.008). (T0)—before the start of orthodontic treatment, (T1) the end of treatment and (T2) 3 months after using additional aligner only at night.

**Figure 12:**
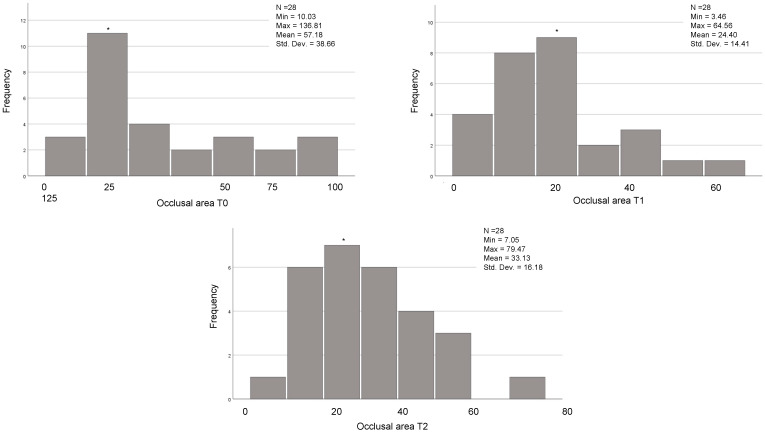
Analysis of occlusal areas at T0, T1 and T2 of the 28 individuals that used AA. Related Sample Friedmand’s Two-Way Test. * Significant difference from oclusal area T0 and T1 (*p* = 0.002) and occlusal area T1 and occlusal area only with night use (*p* = 0.000). (T0)—before the start of orthodontic treatment, (T1) the end of treatment and (T2) 3 months after using additional aligner only at night.

In the 28 cases that completed the proposed orthodontic goals after the use of the additional aligners and the results showed that the occlusal area decreased significantly from T0 to T1 (*p* = 0.002) and increased significantly from T1 to T2 (*p* = 0.000). Regarding posterior occlusal contacts, they decreased from T0 to T1 (*p* = 0.000) and increased significantly from T1 to T2 (*p* = 0.000) (Figure 10). Lastly, the anterior occlusal contacts, significantly decreased from T0 to T1 (*p* = 0.008) (Figure 11). Total occlusal contacts decreased significantly from T0 to T1 (*p* = 0.000) and showed a statistically significant increase from T1 to T2 (*p* = 0.000) (Table 11 and Figure 12).

### 3.10. Case Complexity and Facial Biotype

The results of the relationship between case complexity and facial biotype are presented in Table 12. The facial biotype presenting the highest complex cases percentage was the hyperdivergent. At the end of the first series of CA, 71.4% (n = 15) of the hypodivergent cases were completed, as well as 64.7% (n = 22) of the normodivergent and 63.0% (n = 17) of the hyperdivergent (Table 13). There was no statistically significant relationship between the facial biotype and treatment’s completion after the first set of CA (Table 13).

## 4. Discussion

The growing number of orthodontic treatments performed with CA, [28] in which the occlusal coverage prevents the obtention of natural contacts, has become a crucial research topic in Dentistry, [29] more specifically in the orthodontic community. The presence of the CA material in the interocclusal space may lead to anatomical changes and difficulties that are inherent to the obtention of those contacts and therefore compete against the final goal of CAT [1,7,8,13]. Of the 82 individuals that were selected for this study, only 54 reached the planned orthodontic goals and had their treatment considered complete by the end of the first set of aligners. These 54 individuals then moved on the next phase of the study, which aimed to evaluate occlusal changes 3 months after the end of the first set while using additional CA only at night during this time. In these 54 cases these additional aligners used only at night allowed finishing enhancements and improved occlusal settling. These slight movements are more directed toward improving occlusal contacts. However, given the algorithm created by the Clincheck^®^ software, other minor movements may occur, in order to perform additional fine-tuning details without major clinical significance. The 28 individuals that did not finish their treatment by the end of the first set of CA needed additional aligners to complete their orthodontic treatment goals. In this study we report that 64% of these individuals needed 3 AA in order to complete the treatment. When evaluating the 82 individuals, after completing the orthodontic treatment, independently from using additional aligners to complete the orthodontic treatment, or finishing after the first set of CA, the results showed a statistically significant decrease in the number of occlusal contacts and areas recorded between T0 and T1.

Our reported decrease in the number of occlusal contacts and areas between T0 and T1 corroborates previous published results [8,30,31,32] It is thought that this occurred due to the thickness of two thermoplastic devices which creates a posterior “open-bite”.This is due to their prolonged use between the dental arches, resulting in molar intrusion, [7,33] and altering the number and quality of the existing occlusal contacts, which goes against the final goal of CAT. Horton et al. [34]. also describes a similar significant reduction in the interocclusal contact area after the use of an occlusal-covered appliance (Essix) compared to the use of a Hawley splint (no occlusal-covering). In addition, our results show that using CA at night for 3 months after treatment completion enhances occlusal area, total occlusal contacts, and posterior occlusal contacts do enhance after. We observed this increase whether treatment completion was achieved after one set or with the aid of AA. As suggested by Sultana et al. (2002) [35], this increase could be explained by the fact that during the period following the conclusion of the treatment, when using CA only at night, a functional accommodation of occlusion occurs, leading to an increase in the number of occlusal contacts. According to the results obtained, utilizing a CA only at night for three months, after the completion of the treatment appears to enhance the restitution of the occlusal contacts (area, posterior and total occlusal contacts). Similar observations have been made in other studies where other devices were used [35,36,37].

In the present study, the differences between the number of contacts (anterior and posterior) planned and those effectively obtained at the first set of CA were evident. However, the number of anterior contacts obtained was significantly higher than planned, and the number of posterior contacts obtained was significantly lower. Charalampakis et al., [13] described that the CA thickness promoting a bite-block effect, and the presence of premature contacts in the anterior area are some of the factors that can lead to the loss of posterior contacts during CAT [13]. This study results suggests that a temporary iatrogenic open bite can occur derived from the OT, corroborating what was described in other studies.

Further analysis of occlusal changes obtained at T2 show an increase in recovery of the occlusal contacts and areas. This could be due to the tendency of the posterior teeth to naturally execute relative movements in the vertical direction, through the physiologic eruption process which increases the number of occlusal contacts during the settling phase [35,36]. Previous studies documented that a complete settling requires time [37,38]. Horton et al. [34] showed that most of the settling occurred within the first three months post-treatment, aligning with the presented our results.

Those findings suggest that with careful planning and proper knowledge of the CA system’s limitations and how to counter them, ideal static occlusal objectives can be achieved with clear aligners orthodontic treatment [39].

As reported in the literature, the occlusion was here obtained by intraoral scanning, where both sides scans of both sides, left and right were afterwards superposed. It is important to acknowledge that scanner accuracy varies in terms of fidelity and precision, and it is known that errors may occur due to how the occlusion is collected from the individual. The scanner used in this study was the iTero, which is considered as one of the most reliable intra-oral scanners. Additionally incorporating traditional methods, such as articulating paper, alongside digital methods like the intraoral scanner, allows cross-verification when recording occlusal contacts, permitting visual confirmation of occlusal contact.

28 individuals did not reach their orthodontic goals, and therefore did not complete the treatment by the end of the first set of aligners. We considered pertinent to study the reasons behind this incompletion. To understand the underlying reason for this incompletion, we wanted to study if case complexity could have a possible implication, as well as verify if there were any movements that could be related to this. By the end of the first set of CA, we observed that the anteroposterior corrections, crowding, and the deep bite in these 28 patents were the most frequent movements that contributed to the classification of these cases as of hard or moderate complexity.

In line with what has been described by Djeu et al.’s study [30], our results demonstrated that anteroposterior corrections were the most difficult movements to execute. Furthermore, they reported lower rates of correction of anteroposterior discrepancies with CAT compared to fixed appliances, referring to the need for additional anchorage techniques. In fact, of the twelve cases presenting the need for anteroposterior movement, seven were not concluded after the first set of aligners [30]. Complex distalization movement was the most relevant movement that did not allow the conclusion of the CAT after the first series of aligners. It would be important to consider in complex cases of distalization movements, to place auxiliaries in the first set of aligners [40]. This movement was also considered difficult by Patterson et al. [41] This author considered that distalization is a difficult movement to solve, probably due to the inadequate wearing time assigned to each aligner to perform such movements or to patient compliance. In the present study, crowding appears to be the second most prevalent movement that determined the degree of complexity of the case. Of all the cases of crowding, only one was not corrected after the first set of CA. Other authors reported the same success rate with crowding correction [7,42,43]. To the best of our knowledge, no other studies have evaluated which tooth movements prevented the completion of orthodontic treatment by CA after the end of the first series of aligners.

At T1, of the 28 cases that did not reach the orthodontic goals (8 being classified as complex and 20 being moderate), the most prevalent movements that did not fully occur were the distalization mentioned above, but also severe rotation of the upper central and rotation of the upper lateral incisive. As described to in the literature, distalization movements of 2 to 4 mm already fall within moderate complexity and often require auxiliary techniques and accessories [44,45]. Regarding the second most difficult movement to correct in our sample was the rotation movement. The same difficulty was reported by Simon et al. [46] and Kravitz et al. [47] These authors suggest that thermoplastic appliances tend to lose anchorage and slip due to the presence of few brackets and the round shape of the tooth and that this could explain why the rotation movement is difficult to achieve. In these cases, in order to enhance the success rate of the treatment, the number of CA or the wearing time of each aligner could be increased to reduce the degree of movement per aligner, using additional aligner. Our result showed that the number of additional aligners needed to achieve the desired outcomes is approximately 3, which is in agreement with the study of Arqub et al. [2]. It would be of great interest for orthodontists to keep this in mind while using Clincheck^®^ to plan the treatment since the software cannot plan the mandible dynamics. The human knowledge of the number of ligaments and muscles could influence the treatment’s success [48].

The skeletal feature has already been described in the literature as influencing the bite-block effect [23]. Hypodivergent biotype individuals are associated with greater bite force. Therefore, we expected that they would present fewer posterior contacts due to the generated intrusive forces in the posterior sectors in comparison with the other two biotypes [18,19,21]. However, no statistically significant differences between the number and contact areas between T0 and T1 were measured for any the facial biotypes or between the different facial biotypes. These results suggest that the facial biotype does not directly influence the areas and the number of occlusal contacts obtained at the end of the first set of CA.

When relating the number of planned and obtained posterior contacts (at T1) with the facial biotype, the results suggested that the planning of posterior contacts with CA is more complex in hypodivergent biotype cases. The hyperdivergent biotype cases however had higher median of values, resulting from the difference between the number of anterior contacts planned and obtained at T1. Corroborating our results, Riede et al. [49] concluded that only 60% of the planned occlusal contacts, obtained through the ClinCheck^®^, were effectively attained. To the best of our knowledge, no other studies have related facial biotypes with the number of occlusal contacts planned and obtained through CAT. These findings emphasize the need for orthodontists to consider occlusal contacts in their planning and include overcorrections, which could allow achieving their therapeutic goals to be achieved with as few sets of additional aligners as possible [50].

After studying facial biotype, case complexity, and success rate of CAT, no correlation was found after completing the first set of CA, suggesting that the success rate is independent of those variables.

One of the limitations of this study was the difficulty ensuring the compliance from each individual to wear the CA during the recommended hours. Additionally, and due to the fact that our study sample was a convenience sample did not allow a homogeneous group study, which could be responsible for bias and further discrepancies. Finally, another potential limitation may the intra-oral image recollection, since the practitioner has to ensure that each individual performs a correct occlusion, avoiding incorrect superpositions and errors.

## 5. Conclusions

Our results showed a decrease in the occlusal contacts and area either at the end of the first set of clear aligners or after the use of additional aligners to complete the treatment. Regarding the difference between the planned contacts and those obtained, the posterior contacts obtained after treatment were consistently lower than the programmed ones. On the other hand, the anterior contacts obtained were higher than those planned. Moreover, the results showed that some of the tooth movements necessary to complete the treatment successfully were harder than others. Distalization, rotation, posterior intrusion, and extrusion are some of those movements, and using a CA only at night increases occlusal contact recovery after finishing the treatment.

## Figures and Tables

**Table 1:** Inclusion and exclusion criteria for the study.

Inclusion Conditions
Individuals with entire definitive dentition are guaranteed to undergo orthodontic treatment with clear aligners;
Must already have performed all the first series of orthodontic treatments with all clear aligners used;
Individuals with available and complete cephalometric analysis.
Exclusion conditions
Individuals whose occlusal records were incomplete;
Individuals with cognitive or neurological alterations, with identified syndromes, a history of head and neck trauma and/or tumors, and metabolic disorders that affect the joints and/or muscles;
Individuals who were being treated with anti-inflammatory drugs, analgesics, or psychiatric medication;
Individuals who had odontogenic pain or periodontal problems.

**Table 2:** Descriptive statistics referring to continuous variables.

	Average	SD	Median	Minimum	Maximum
Occlusal Area T0	54.13	38.54	41.56	3.40	136.81
Occlusal Area T1	25.25	20.41	21.51	1.83	142.86
Anterior Contacts T0	3.57	2.19	4.0	0	6
Anterior Contacts T1	5.04	2.38	5.0	0	8
Posterior Contacts T0	7.00	1.59	8.0	1	8
Posterior Contacts T1	3.51	1.84	4.0	0	6
Anterior Programmed Contacts T0	0.63	1.19	0	0	6
Posterior Programmed Contacts T0	7.61	0.90	8.0	5	10
Anterior Programmed Contacts T1	0.76	0.97	0	0	4
Posterior Programmed Contacts T1	7.53	1.42	8.0	0	9

**Table 3:** Comparison of occlusal areas, anterior and posterior contacts at T0 and T1.

		N	Mean Rank	Z	*p*
Occlusal Area T1—Occlusal Area T0	Negative Ranks	55 ^a^	41.71	−5.50	<0.001
	Positive Ranks	17 ^b^	19.65		
	Ties	10 ^c^			
No. of Posterior Contacts T1 No. of Posterior Contacts T0	Negative Ranks	73 ^d^	41.23	−7.39	<0.001
	Positive Ranks	5 ^e^	14.30		
	Ties	4 ^f^			
No. Anterior Contacts T1 No. Anterior Contacts T0	Negative Ranks	27 ^g^	27.78	−3.70	<0.001
	Positive Ranks	49 ^h^	44.41		
	Ties	6 ^i^			

^a^ Occlusal Area T1 < Occlusal Area T0; ^b^ Occlusal Area T1 > Occlusal Area T0; ^c^ Occlusal Area T1 = Occlusal Area T0; ^d^ No. of Posterior Contacts T1 < No. of Posterior Contacts T0; ^e^ No. of Posterior Contacts T1 > No of Posterior Contacts T0; ^f^ No.of Posterior Contacts T1 = No of Posterior Contacts T0; ^g^ No. of Anterior Contacts T1 < No. of Anterior Contacts T1; ^h^ No. of Anterior Contacts T1 > No. of Anterior Contacts T1; ^i^ No. of Anterior Contacts T1 = No. of Anterior Contacts T1. Wilcoxon Signed Test.

**Table 4:** Comparison between number of programmed and obtained, anterior and posterior contacts after the first set of CA.

		N	Mean Rank	Z	*p*
No. of Posterior Contacts T1—No. of Programmed Posterior Contacts	Negative Ranks	60 ^a^	36.32	−6.52	<0.001
	Positive Ranks	7 ^b^	14.14		
	Ties	15 ^c^			
No. of Anterior Contacts T1—No. of Programmed Anterior Contacts	Negative Ranks	3 ^d^	38.26	−7.48	<0.001
	Positive Ranks	77 ^e^	18.50		
	Ties	2 ^f^			

^a^ No. of posterior contacts T1 < No of scheduled posterior contacts; ^b^ No. of posterior contacts T1 > No. of scheduled posterior contacts; ^c^ No. of posterior contacts T1 = No. of scheduled posterior contacts; ^d^ No. of anterior contacts T1 < No. of anterior contacts scheduled; ^e^ No. of anterior contacts T1 > No. of anterior contacts scheduled; ^f^ No. of anterior contacts T1 = No. of anterior contacts scheduled. (Wilcoxon Signed Test).

**Table 5:** Comparison of the occlusal area and the number of anterior and posterior contacts at T0 and T1, according to facial biotype.

	Normodivergent (n = 34)	Hyperdivergent (n = 27)	Hypodivergent (n = 21)	H	*p*	Effect Size
Occlusal Area T0	46.02 [21.61–92.22]	41.28 [26.75–85.39]	41.30 [19.83–81.35]	0.323	0.851	[−0.1; 0.93]
Occlusal Area T1	21.82 [12.79–32.80]	16.23 [8.11–24.97] *	28.24 [15.51–40.91] *	7.59	0.022	[−0.08; 0.18]
Anterior Contacts T0	4.5 [2.0–6.0]	4.0 [2.0–6.0]	4.0 [1.0–5.5]	1.45	0.778	[−0.1; 0.01]
Anterior Contacts T1	5.5 [4.0–8.0] **	4.0 [2.0–5.0] **	6.0 [3.0–8.0]	6.49	0.037	[−0.09; 0.16]
Posterior Contacts T0	8.0 [7.0–8.0]	8.0 [6.0–8.0]	7.0 [6.0–8.0]	2.26	0.323	[−0.11; 0.07]
Posterior Contacts T1	4.0 [2.0–5.0]	4.0 [1.0–4.0]	4.0 [1.0–5.0]	2.26	0.324	[−0.11; 0.06]

Data summarized as the median and interquartile range (IQR); *p*-value derived from the Kruskal-Wallis test and Dunn’s test. * Significant difference between the hyperdivergent and hypodivergent groups (*p* = 0.031); ** Significant difference between the hyperdivergent and normodivergent groups (*p* = 0.044). (Kruskal-Wallis test).

**Table 6:** Comparison between posterior, anterior, and total contacts at the T0, T1 and T2 in the group that achieved the orthodontic goals after the first set of CA.

	Median (IQR)	*χ* ^2^	*p*
Occlusal Area T0	48.42 [21.15; 92.21] *	19.16	<0.001
Occlusal Areal T1	21.8 [10.35; 36.78] *		
Occlusal Area T2	34.93 [23.67; 48.20] *		
Posterior Contacts T0	8.0 [5.75; 8.0] **	37.93	<0.001
Posterior Contacts T1	5.0 [3.0; 7.0] **		
Posterior Contacts T2	7.0 [6.0; 8.0] **		
Anterior Contacts T0	4.0 [1.0; 6.0]	0.44	0.802
Anterior Contacts T1	4.0 [2.0; 5.25]		
Anterior Contacts T2	4.0 [2.75; 5.25]		
Total Contacts T0	11.0 [8.0; 13.0] ***	14.32	<0.001
Total Contacts T1	8.0 [6.0; 10.25] ***		
Total contacts T2	11.0 [9.0; 12.0] ***		

Data summarized as the median and interquartile range (IQR); *p*-value derived from the Friedman Related-Samples Friedman’s Two-way of variance ranks; * Significant difference from occlusal area T0 and T1 (*p* < 0.001) and occlusal area T1 and occlusal area only with night use (*p* = 0.001); ** Significant difference from posterior contacts T0 and T1 (*p* < 0.001) and T1 and between posterior contacts T1 and posterior contacts only with night use (*p* < 0.001); *** Significant difference from the total contacts T0 and T1 (*p* = 0.014) and Total contacts T1 and total contacts only with night use (*p* = 0.004.

**Table 7:** Movements that justify the complexity of the cases.

Type of Movement	n (%)
AP Correction	12 (20.3)
Crowding	8 (13.6)
Deep bite	5 (8.5)
Rotations	4 (6.8)
Narrow arches	4 (6.8)
Narrow arches and AP correction	4 (6.8)
Anterior open bite	4 (6.8)
Crowding AP correction	3 (5.1)
AP correction, rotations	3 (5.1)
Spacing	3 (5.1)
Crowding, rotations	2 (3.4)
Crowding, narrow arch	1 (1.7)
Crowding, narrow arch, posterior crossbite	1 (1.7)
AP correction, alignment, posterior crossbite	1 (1.7)
AP correction, spacing, narrow arches	1 (1.7)
Anterior open bite, AP correction	1 (1.7)
Rotations, AP correction, deep bite	1 (1.7)
Rotations, crowding, AP correction, deep bite	1 (1.7)

AP—Antero-posterior.

**Table 8:** Movement responsible for not completing the treatment at T1.

Movement	n (%)
Distalization	7 (25.0)
ICS-ICI rotation	4 (14.3%)
Posterior extrusion	3(10.7)
Posterior intrusion	3 (10.7)
Quadrant expansion	3 (10.7)
ILS rotation	2 (7.1)
Sup-Inf. Rotation Can/PM	2 (7.1)
Anterior intrusion	2 (7.1)
Anterior extrusion	1 (3.6)
Crowding	1 (3.6)

ICS—Upper central incisor; ICI—Lower central incisor; ILS—Upper lateral incisor; Can—Canine; PM—Pre-Molar.

**Table 9:** Degree of the movements responsible for not completing the treatment at T1.

Movement												
		Anterior Extrusion	Anterior Intrusion	Crowding	Distalization	Expansion per Quandrant	Posterior Extrusion	Posterior Intrusion	Rotation Can/PM Sup_Inf	Rotation ILS	Rotation-ICS-ICI	Total
Degree of the movement	<1 mm	0	0	0	0	0	0	2	0	0	0	2
	<2.5 mm	0	2	0	0	0	0	0	0	0	0	2
	<2 mm	0	0	0	0	2	0	0	0	0	0	2
	>1 mm	0	0	0	0	0	3	1	0	0	0	4
	>3 mm	1	0	0	0	0	0	0	0	0	0	1
	0–30°	0	0	0	0	0	0	0	0	1	0	1
	0–40°	0	0	0	0	0	0	0	0	0	3	3
	0–45°	0	0	0	0	0	0	0	1	0	0	1
	2–4 mm	0	0	0	7	1	0	0	0	0	0	8
	30–40°	0	0	0	0	0	0	0	0	1	0	1
	40–50°	0	0	0	0	0	0	0	0	0	1	1
	45–55°	0	0	0	0	0	0	0	1	0	0	1
	6–8 mm	0	0	1	0	0	0	0	0	0	0	1
Total		1	2	1	7	3	3	3	2	2	4	28

ICS—Upper central incisor; ICI—Lower central incisor; ILS—Upper lateral incisor; Can—Canine; PM—Pre-Molar.

**Table 10:** The number of additional aligners needed to finish the treatment.

No. of Additional Aligners	n (%)
1 Additional aligners	4 (14.3)
2 Additional aligners	6 (21.4)
3 Additional aligners	18 (64.3)
Total	28 (100)

**Table 11:** Comparison between posterior, anterior, and total contacts at the T0, T1 and T2, of the 28 individuals who completed orthodontic treatment using additional aligners.

	Median (IQR)	*χ* ^2^	*p*
Occlusal Area T0	39.21 [28.53; 90.39] *	19.79	<0.001
Occlusal Areal T1	22.89 [14.00; 33.35] *		
Occlusal Area T2	31.40 [19.79; 43.61] *		
Posterior Contacts T0	8.0 [7.0; 8.0] **	44.31	<0.001
Posterior Contacts T1	5.0 [3.0; 6.0] **		
Posterior Contacts T2	7.5 [6.25; 8.0] **		
Anterior Contacts T0	5.0 [2.0; 6.0] ***	11.68	0.003
Anterior Contacts T1	2.0 [1.0; 2.0] ***		
Anterior Contacts T2	2.0 [2.0; 3.0]		
Total Contacts T0	12.0 [9.25; 13.75] ****	43.33	<0.001
Total Contacts T1	6.0 [5.0; 7.0] ****		
Total contacts T2	10.0 [9.0; 10.0] ****		

Data summarized as the median and interquartile range (IQR); *p*-value derived from the Friedman Related-Samples Friedman’s Two-way of variance ranks * Significant difference from oclusal area T0 and T1 (*p* = 0.002) and occlusal area T1 and occlusal area only with night use (*p* = 0.000) ** Significant difference from posterior contacts T0 and T1 (*p* = 0.000) and T1 and between posterior contacts T1 and posterior contacts only with night use (*p* = 0.000); *** Significant difference from anterior contacts T0 and T1 (*p* = 0.008) **** Significant difference from the total contacts T0 and T1 (*p* = 0.000) and Total contacts T1 and total contacts only with night use (*p* = 0.000).

**Table 12:** Relationship between facial biotype and case complexity.

Case Complexity						
		Complex	Moderate	Simple	Total	*p*
		n (%)	n (%)	n (%)	n (%)	
Facial Biotype	Hyperdivergent	6 (2.2)	13 (48.1)	8 (29.6)	27 (100.0)	0.71
	Hypodivergent	2 (9.5)	12 (57.1)	7 (33.3)	21 (100.0)	
	Normodivergent	5 (14.7)	21 (61.8)	8 (3.5)	34 (100.0)	
Total		13	46	23	82	

Fisher’s exact test.

**Table 13:** Relationship between facial biotype and completion of CAT at T1.

Completed Cases at the Enf of the First Set of CA					
		YES	NO	Total	*p*
		n (%)	n (%)	n (%)	
Facial Biotype	Hyperdivergent	10 (37.0)	17 (63.0)	27 (100.0)	0.87
	Hypodivergent	6 (28.6)	15(71.4)	21 (100.0)	
	Normodivergent	12 (35.3)	22 (64.7)	34 (100.0)	
Total		28	46	82	

Fisher’s exact test.

## Data Availability

The authors declare that the data supporting the findings of this study are available within the article.
